# Psychometric properties of the Coronavirus Anxiety Scale based on Classical Test Theory (CTT) and Item Response Theory (IRT) models among Chinese front-line healthcare workers

**DOI:** 10.1186/s40359-023-01251-x

**Published:** 2023-08-07

**Authors:** Dongmei Zhang, Congzhi Wang, Ting Yuan, Xiaoping Li, Liu Yang, Anle Huang, Jing Li, Mingming Liu, Yunxiao Lei, Lu Sun, Jing Zhang, Lin Zhang

**Affiliations:** 1 School of Nursing, Wannan Medical College, 22 Wenchang West Road, Higher Education Park, Anhui Province Wuhu City, P.R. China; 2 Nursing Department, the People’s Hospital of Yingshang, 566 Ganluo Road, Chengbei New District, Yingshang County, Anhui Province Fuyang, P.R. China

**Keywords:** Anxiety, Healthcare workers, COVID-19, Reliability, Validity

## Abstract

**Background:**

Since March 2022, the COVID-19 epidemic has rebounded widely and frequently in China. Healthcare workers have faced grand challenges such as soaring COVID-19 patients, being busy with the nucleic acid screening of all the populations in the epidemic areas every day, and testing positive for COVID-19, all of which contributed to anxiety easily according to the Conservation of Resources theory. However, anxiety among healthcare workers is not only associated with personal health but also adversely affects the quality of health services. Therefore, it is crucial to search for suitable tools to monitor the anxiety related to COVID-19 among healthcare workers. The current study aimed to test the Coronavirus Anxiety Scale (CAS) in Chinese healthcare workers.

**Methods:**

The current study employed a cross-sectional design. The CAS was translated into Chinese. Then, according to Classical Test Theory (CTT) and Item Response Theory (IRT) models, the psychometric properties of the Chinese version were measured among 811 healthcare workers.

**Results:**

The split‐half reliability was 0.855. The Cronbach’s α coefficient was 0.895. The retest coefficient was 0.901 with 10 days as the retest interval. The content validity index was 0.920. In exploratory factor analysis, one common factor was extracted and explained 72.559% of the total variance. All item load values on the common factor ranged from 0.790 to 0.885, and the communality of each item ranged from 0.625 to 0.784. With confirmatory factor analysis, the single factor model showed an excellent goodness-of-fit, chi-square/degree of freedom (*χ*^*2*^/*df*) = 3.339, goodness of fit index (GFI) = 0.992, adjusted goodness of fit index (AGFI) = 0.975, root-mean-square error of approximation (RMSEA) = 0.054, root mean square residual (RMR) = 0.005, incremental fit index (IFI) = 0.967, Tucker-Lewis index (TLI) = 0.932, and comparative fit index (CFI) = 0.966. The multiple-group confirmatory factor analysis revealed the invariance measuring anxiety of COVID-19 was in similar ways across ages, hospital degrees, and professional titles. With convergent validity, the CAS was positively correlated with post-traumatic stress disorder (*r* = 0.619, *P* < 0.001), fear of COVID (*r* = 0.550, *P* < 0.001), and depression (*r* = 0.367,* P* < 0.001). According to IRT models, the results showed that all item discrimination parameters were higher than 1.70 and difficulty parameters ranged from 1.13 to 2.83.

**Conclusion:**

The Chinese version of CAS has good psychometric properties in healthcare workers after China adjusted the COVID-19 management measures during the COVID-19 Omicron epidemic, and can be used for assessing the anxiety associated with COVID-19 in Chinese healthcare workers.

## Background

The coronavirus disease 2019 (COVID-19) pandemic has swept the world for over three years and is still from its conclusion [1–3], and the emergency of Omicron variants has sparked the fifth wave of the outbreak worldwide [4, 5]. On December 2021, China officially announced adopting a ‘‘dynamic zero-COVID policy”. The policy stipulates that immediate and effective measures should be adopted when one case occurs. Since March 2022, the epidemic has rebounded widely and frequently in China [6]. The outbreak was triggered by highly contagious Omicron as the prevalent strain [7]. Additionally, China owned a high population density, which led to the risk of transmission easily. These have given rise to the rapid and large-scale spread of the outbreak [8]. Accordingly, healthcare workers, who are the main force fighting against the pandemic, faced grand physical and mental pressure such as soaring COVID-19 patients, being busy with the nucleic acid screening of all the populations in the epidemic areas every day, and testing positive for COVID-19 [6], all of which contributed to anxiety easily [9]. Healthcare workers have reported a high prevalence of anxiety, with total prevalence ranging from 23.2% to 67.7% during the COVID-19 pandemic and 50.4% showed substantial anxiety symptoms [10–12]. Compared with other professional groups who directly contacted clients during COVID-19, healthcare workers owned the highest anxiety [13].

The Conservation of Resources theory (COR) [14] is often employed to study major and traumatic stress in crises. COR theory [14] points out people make efforts to gain, retain, and preserve resources that they most value. They feel stressed such as anxiety, when threatened with resource loss, actually lose resources, or resources cannot be recaptured. Additionally, resource loss is considered as having more influence than resources obtained following a crisis. Resources loss was strongly associated with mental distress levels during the pandemic [15]. Front-line healthcare workers are under multiple stressors, such as extremely burdensome and dangerous working conditions, isolation, and so on, which can contribute to resources (mental resources, material resources, and social resources) loss and trigger anxiety easily [16]. However, anxiety among healthcare workers is not only associated with personal health but also adversely affects the quality of health services. Rightfully so, their anxiety deserves more attention. Also, it is crucial to seek for appropriate tools to monitor the anxiety of COVID-19 in healthcare workers.

The Coronavirus Anxiety Scale (CAS) was developed by Lee et al. in the United States [17]. It is a parsimonious and robust tool for assessing anxiety of COVID-19. Subsequently, the CAS has been validated in several languages and corroborated satisfying psychometric properties in different populations, such as the Spanish version (274 Peruvian older adults) [18], Colombian version (421 Colombian adults) [19], Arabic version (237 of 18 to 58 years old adults) [20], Korea version (329 adults) [21], Bangladesh version (737 adults) [22], Slovak version (743 adults) [23], Chinese version (2,116 adults) [24], Latin American version (5196 participants from twelve Latin American countries) [25], and so on. In these different versions, the CAS maintained its original single-factor structure. Moreover, all of the CAS items (five items) were internally consistent, with Cronbach’s α coefficients ranging from 0.80 to 0.95 in those different versions [17, 19–26].

Significantly, most of the previous studies employed the Classical Test Theory (CTT) to assess the psychometric properties of the CAS. However, the CTT doesn’t assess the symptomatology of anxiety throughout the range of anxiety severity. Item Response Theory (IRT) models can make up for this deficiency [27]. IRM employs item responses to create a linear scale that represents ‘less’ to ‘more’ of a characteristic or latent variable [27]. Therefore, the relationship between respondent location and item location on the scale of that latent variable can be compared directly [28].

However, so far, IRT models have not been employed to assess the CAS in China. Additionally, the Chinese version of the CAS has not been tested among healthcare workers. If there is no psychometric proof relating to the usage of a scale for a certain population, it will restrict the scale being used to evaluate this population and then affects clinical intervention for the population. Also, it is necessary to assess the psychometric properties in new situations because tools that have been validated in the past may no longer work now because of ongoing changes [29, 30].

Given that, this study cross-culturally validated the CAS in Chinese healthcare workers, employing CTT and IRT models together. Firstly, the reliability of the CAS was calculated by split-half reliability, Cronbach’s α coefficient, item-total correlation, and retest reliability. Secondly, the validity of the CAS was assessed by content validity and construct validity. Thirdly, discrimination and difficulty were estimated for each item based on IRT models. Simultaneously, we hypothesized that all the results were good, which indicated the CAS had good psychometric properties.

## Methods

### Translation procedure

We took several steps as follows according to translation criteria [31, 32]. Firstly, the English version of the CAS was translated into Chinese by two translators who were bilingual professionals. Secondly, the Chinese version was translated back into English by another two bilingual professional translators. Thirdly, the cultural and linguistic equivalence of the CAS was assessed by three medical experts and two psychology experts. Fourthly, the Chinese version was used to test 20 healthcare workers (10 nurses and 10 doctors). Based on the healthcare workers’ feedback, the Chinese version of CAS was revised again and formed initially (Table 1).

**Table 1:** The coronavirus anxiety scale (English version and Chinese version)

Item	Item content	Score
Item 1	I felt dizzy, lightheaded, or faint, when I read or listened to news about the coronavirus	0 1 2 3 4
	当我看到或听到有关新冠的消息时, 我就感到天旋地转、头昏眼花或快要晕倒了。	
Item 2	I had trouble falling or staying asleep because I was thinking about the coronavirus	0 1 2 3 4
	因为我一直在想有关新冠的事情, 所以我很难入睡或难以沉睡。	
Item 3	I felt paralyzed or frozen when I thought about or was exposed to information about the coronavirus	0 1 2 3 4
	当我想到或接触到有关新冠的信息时, 我就感到不能动弹, 像被冻僵了一样。	
Item 4	I lost interest in eating when I thought about or was exposed to information about the coronavirus	0 1 2 3 4
	当我想到新冠或接触到有关的信息时, 我就没胃口进食了。	
Item 5	I felt nauseous or had stomach problems when I thought about or was exposed to information about the coronavirus	0 1 2 3 4
	当我想到新冠或接触到有关的信息时, 我就觉得恶心想吐或肠胃不适。	

### Participants and procedures

The target population encompassed healthcare workers from four hospitals in Yingshang County, Anhui province, where the outbreak was triggered by highly contagious Omicron in the whole city. According to Kendall’s criterion, tenfold the number of total items and added no fewer than 10% [33, 34], a sample size of no less than 374 was figured up since the total items of four scales in the current study are 34. The inclusion criteria are as follows, (i) having participated in fighting against COVID-19; (ii) full-time healthcare worker in a hospital; (iii) having not participated in the pilot test; (iv)volunteering to take part in the present study. Questionnaire star was applied to issuing the questionnaires. Before data collection, we obtained informed consent from all participants. Once participants agree, they can access the questionnaire-filling interface and click on their choice. Finally, 853 questionnaires were received from May 20 to 31, 2022, during the Omicron variants epidemic, of which 811 (95.08%) were qualified questionnaires. Moreover, 20 randomly selected healthcare workers were invited to complete the questionnaires again 10 days later. The average time to finish this questionnaire took approximately 275 s.

### Instruments

#### Sociodemographic information

According to the relevant studies [17, 35–37], sociodemographic information was collected, including sex, age, occupation, professional title, years of working, and hospital degree.

#### Coronavirus Anxiety Scale (CAS)

The CAS is a 5-item, single-factor tool [17]. The CAS employs a 5-point Likert scale with response choices ranging from “not at all over the last 2 weeks” to “nearly every day over the last 2 weeks”. The total scores of the scale range from 0 to 20. A CAS score of 9 is the optimal cutoff value for screening purposes in the original scale. The higher the total scores of CAS represent the higher anxiety of COVID-19. The Cronbach’s α coefficient of CAS is 0.93 in Lee et al.’s study [17].

#### Posttraumatic Stress Disorder Checklist for DSM-5 (PCL-5)

The PCL-5 contains 20 items that can be divided into four subscales [38]. The items are rated on a 5-point Likert-type scale from 0 (not at all) to 4 (extremely). The total scores of PCL-5 range from 0 to 80. The Cronbach’s α coefficient is 0.94 [38]. Cheng et al. [36] have corroborated that PCL-5 owned excellent psychometric properties in China. The Cronbach’s α coefficient of PCL-5 is 0.91 in Cheng et al.’s study [36], and 0.932 in this study.

#### Fear of COVID-19 Scale (FCV-19S)

FCV-19S was developed by Ahorsu et al. [39]. It is a seven-item self-reporting measurement tool for evaluating the degree of fear of COVID-19. Its items are rated on a 5-point Likert-type scale. The answers are ranging from 1 (strongly disagree) to 5 (strongly agree). The total scores of FCV-19S range from 7 to 35. The higher the total scores represent the worse fear of COVID-19. The Cronbach's α coefficient of FCV-19S is 0.820 in Ahorsu et al.’s study [39]. Feng et al. [40] have validated the Chinese version owns excellent psychometric properties. The Cronbach’s α coefficient of FCV-19S is 0.924 in Feng et al.’s study [40], and 0.927 in the present study.

#### Patient Health Questionnaire-2 (PHQ-2)

The PHQ-2 is commonly used as a parsimonious depression screening measure with only two items [41]. For each item, the response options are on a 4-point Likert-type scale ranging from “not at all” (0) to “almost every day” (3). Thus, the total scores of PHQ-2 range from 0 to 6. A PHQ-2 score of 3 is the optimal cutoff value for screening purposes in the original scale. The Chinese version of PHQ-2 has been corroborated well psychometric properties, with Cronbach’s α coefficient ranging from 0.727 to 0.785 [42]. The Cronbach’s α coefficient of PHQ-2 is 0.804 in this study.

### Statistical analysis

SPSS 25.0, AMOS 23.0, and R 4.3.0 were employed to analyze the statistics. The reliability of the CAS was calculated by split-half reliability, Cronbach’s α coefficient, item-total correlation, and retest reliability.

The content validity index (CVI) was employed to assess the content validity of the CAS and was examined by three medical experts and two psychology experts. The experts were asked to score each CAS item on its relevance using a 4-item Likert-type format, ranging from 1 (not relevant) to 4 (highly relevant) [43]. CVI was rated as good when an item content validity index (I-CVI) and the average of all the I-CVIs of the individual items (S-CVI_Ave_) were not less than 0.78 and 0.90, respectively [43].

Exploratory factor analysis (EFA), confirmatory factor analysis (CFA), cross-validation, and construct convergent validity were employed to test the construct validity. With regard to EFA, principal component analysis (PCA) with varimax rotation was employed. With EFA, the criteria for the load value of each item is not less than 0.40 on the common factor, and the additive contributing rate of the extracted common factors is higher than 40% [44]. In CFA, eight indices were applied to evaluate model fit, including chi-square/degree of freedom (*χ*^*2*^/*df*), goodness of fit index (GFI), adjusted goodness of fit index (AGFI), root-mean-square error of approximation (RMSEA), root mean square residual (RMR), incremental fit index (IFI), Tucker-Lewis index (TLI), and comparative fit index (CFI). The recommended value of GFI, AGFI, IFI, TLI, and CFI are all higher than 0.90, *χ*^*2*^/*df* is less than 5, the RMSEA should be less than 0.08, and RMR is less than 0.05 [45]. Also, the Bayesian information criterion (BIC), whose value is lower indicating a better model fit [45], was used to assess which fit is better in single dimension and two dimensions. As for cross-validation, multiple-group confirmatory factor analysis (MGCFA) was utilized to evaluate measurement invariance across demographic groups. Because the Chi-square values were sensitive to the size of the sample, the invariance was mainly assessed by model goodness-of-fit and the change of goodness-of-fit indexes [46]. The criteria for the change of RFI value, TLI value, CFI value, and RMSEA are all less than 0.010 [46, 47]. The construct convergent validity was measured by correlations between the CAS and PTSD, Fear of COVID-19, and depression, because these center on trauma-related reactions, such as mental and somatic symptoms relating to disorders [17]. Also, past studies have suggested there were significant interactions among anxiety, PTSD, fear, and depression [48–50]. In the correlation analysis, *P* < 0.05 was considered statistically significant and the Bonferroni-corrected significance was determined at *P* < 0.017.

Finally, IRT models were employed to assess the CAS. Akaike information criterion (AIC) and BIC, whose values are lower indicating a better model fit [51], were used to check better fit Graduated Response Model (GRM) and Generalized Partial Credit Model (GPCM). In the current study, the AIC and BIC values of GPCM were 2820 and 2938, while the AIC and BIC values of GRM were 3039 and 3156, respectively. Therefore, the GPCM was employed since it had a better model fit. For each item, the discrimination parameters (*a*) and difficulty parameters (β) were estimated. The criterion for the *a* value is as follows: 1.35 ~ 1.69 = high; > 1.70 = very high [52]. As for difficulty, the criterion is that β values range from -3 to 3 and need not increase monotonically because of the separability of the parameters [53]. Additionally, item information Curves (IIC) and total (scale) information Curves (TIC) were measured. The larger the area covered under the IIC, the item can provide a more precise estimation of anxiety about COVID-19 [54].

### Ethical considerations

The study was approved by the ethical committee of the College of Nursing of Wannan Medical College (20220004). Before the statistics collection, we obtained informed consent from all participating healthcare workers.

## Results

### Descriptive statistics

Of the participating 811 healthcare workers, the ages ranged from 19 to 65 years, with an average of (31.90 ± 7.82). Most of them were females (606, 74.72%), nurses (536, 66.09%), juniors (574, 70.78%), and coming from secondary hospitals (495, 61.04%). Their years of working ranged from 1 to 40 years, with an average of (9.47 ± 7.99) (Table 2).

**Table 2:** Frequency distribution of sociodemographic characteristics (*n* = 811)

Variables	Groups	*n*	%/$\overline{\mathbf{X} }\pm S$
Sex	Male	205	25.28
	Female	606	74.72
Age (years)	19–65	811	31.90 ± 7.82
Occupation	Nurse	536	66.09
	Doctor	275	33.91
Professional title	Junior	574	70.78
	Intermediate grade	182	22.44
	Senior title	55	6.78
Years of working	1 ~ 40	811	9.47 ± 7.99
Hospital degree	Secondary hospital	495	61.04
	Tertiary hospital	316	38.96

Secondary hospital: mainly provides comprehensive medical services

Tertiary hospital: mainly provides high-level specialized medical services

### Reliability

The split‐half reliability was 0.855. The Cronbach’s α coefficient of the CAS was 0.895. Moreover, Cronbach’s α coefficients ranged from 0.862 to 0.899 when each item was removed, which were somewhat below 0.895 except for item 2 (0.899). The corrected item-total correlations ranged from 0.683 to 0.799. The retest coefficient was 0.901 with 10 days as the retest interval.

### Validity

#### Content validity

As shown in Table 3, the I-CVI of the CAS ranged from 0.800 to 1.000 and the S-CVI_Ave_ was 0.920.

**Table 3:** The content validity of the CAS

**Item**	**Number giving a rating of 3 or 4**	**I-CVI**	S-CVI_Ave_
Item 1	4	0.800	0.920
Item 2	5	1.000	
Item 3	4	0.800	
Item 4	5	1.000	
Item 5	5	1.000	

*CAS* Coronavirus anxiety scale

#### Face validity

No major suggestions were given by the healthcare workers. Specifically, a few minor suggestions were offered to improve the clarity of the expression and to correct language errors in Chinese culture, such as “coronavirus” being replaced by “coronavirus disease 2019”.

#### Construct validity

##### Exploratory factor analysis

A Kaiser–Meyer–Olkin (KMO) value of 0.889 and a Bartlett spherical test value of 2509.069 (*df* = 10,* p* < 0.001) in the EFA, showed that the factor analysis was suitable [33]. One common factor was extracted and explained 72.559% of the total variance. All item loading values on the common factor ranged from 0.790 to 0.885. The communality coefficients of items ranged from 0.625 to 0.784 (Table 4).

**Table 4:** Factor loading and communality of each item in CAS (*n* = 811)

Item	Factor loadings	Communalities
Item 3	0.885	0.784
Item 5	0.879	0.772
Item 4	0.867	0.751
Item 1	0.835	0.697
Item 2	0.790	0.625

*CAS* Coronavirus anxiety scale

##### Confirmatory factor analysis

**Fig. 1:**
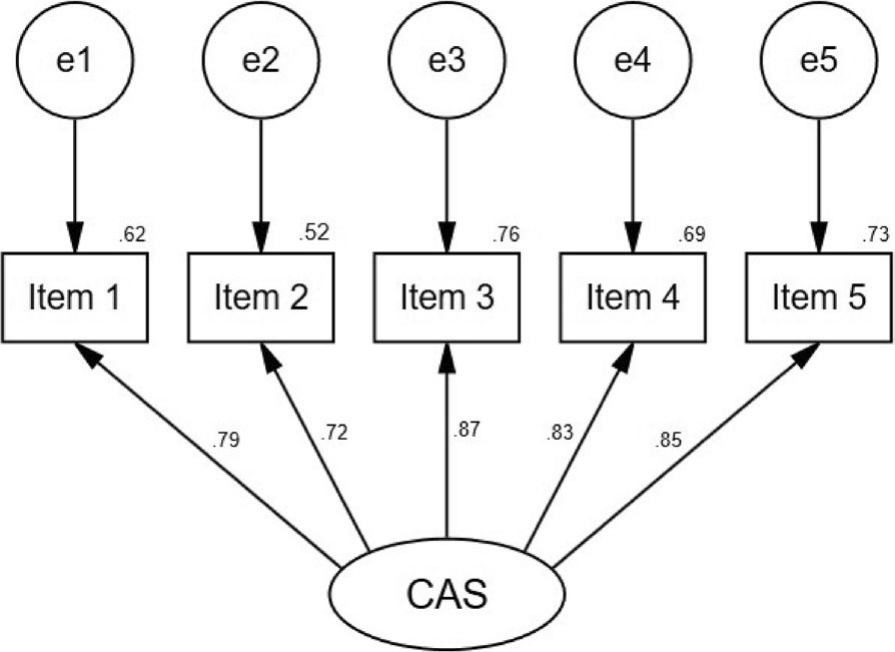
Single-factor structural model of CAS (*n* = 811)

With CFA of the CAS, the unidimensional model revealed an excellent goodness-of-fit, *χ2*/*df* = 3.339, GFI = 0.992, AGFI = 0.975, RMSEA = 0.054, RMR = 0.005, IFI = 0.967, TLI = 0.932, CFI = 0.966 (Fig. 1). Also, A BIC value was 83.676 in the single dimension and 86.792 in the two dimensions, generally considered the single dimension to own a better model fit.

##### Cross-validation

In the cross-validation of the CAS, the results showed invariance between secondary hospitals and tertiary hospitals. Specifically, there was excellent model goodness-of-fit, *χ*^*2*^ = 25.517, *df* = 10, *χ*^*2*^/*df* = 2.552, CFI = 0.994, and RMSEA = 0.044. Simultaneously, there was no significant change in RFI (ΔRFI = 0.001), TLI (ΔTLI = 0.001), CFI (ΔCFI = 0.004), RMSEA (ΔRMSEA = 0.002). The results also showed invariance between juniors and non-juniors. Specifically, there was good model goodness-of-fit, *χ*^*2*^ = 38.721*, df* = 8*, χ*^*2*^/*df* = 4.840, CFI = 0.988, and RMSEA = 0.069. Simultaneously, there was no significant change in RFI (ΔRFI = 0.004), TLI (ΔTLI = 0.004), CFI (ΔCFI) = 0.003, and RMSEA (ΔRMSEA = 0.005). Moreover, the results showed invariance between the age of < 30 and ≥ 30. Specifically, there was acceptable model goodness-of-fit, *χ*^2^ = 53.294*, df* = 10*, χ*^*2*^/*df* = 5.329, CFI = 0.983, and RMSEA = 0.073. There was no significant change in RFI (ΔRFI = 0.008), TLI (ΔTLI = 0.008), and RMSEA (ΔRMSEA = 0.008).

##### Construct convergent validity

As for the construct convergent validity of the CAS, we tested the correlations between the CAS and the PCL-5, the FCV-19S, and the PHQ-2. The results revealed that the CAS was positively correlated with the PCL-5 (*r* = 0.619, *P* < 0.001), the FCV-19S (*r* = 0.550, *P* < 0.001), and the PHQ-2 (*r* = 0.367,* P* < 0.001). Also, all the correlations were still statistically significant using Bonferroni correction for multiple testing (*P* < 0.017).

### The analyses of sociodemographic characteristics

Mann–Whitney *U* test revealed that tertiary hospitals owned significantly higher CAS scores than secondary hospitals (*Z* = 3.752, *P* < 0.001). Kruskal–Wallis test showed that intermediate grades had significantly higher CAS scores than junior (*H* = 4.672, *P* < 0.001) and senior titles (*H* = 4.672, *P* = 0.003). There was no significant difference between junior and senior titles, as well as between males and females.

### Item response theory models

The results of EFA and CFA corroborated the unidimensionality and local independence, which illustrated a GPCM can be used. Table 5 showed that all item discrimination parameters ranged from 2.08 to 3.85. As for difficulty, the parameters ranged from 1.13 to 2.83.

**Table 5:** Discrimination and difficulty parameters for scale items (*n* = 811)

Item	*a*	β_1_	β_2_	β_3_	β_4_
Item 1	2.72	1.68	2.48	2.83	2.43
Item 2	2.08	1.13	2.49	2.20	2.29
Item 3	3.85	1.76	2.53	2.51	2.64
Item 4	3.24	1.39	2.49	2.62	2.58
Item 5	3.82	1.75	2.51	2.66	2.58

**Fig. 2:**
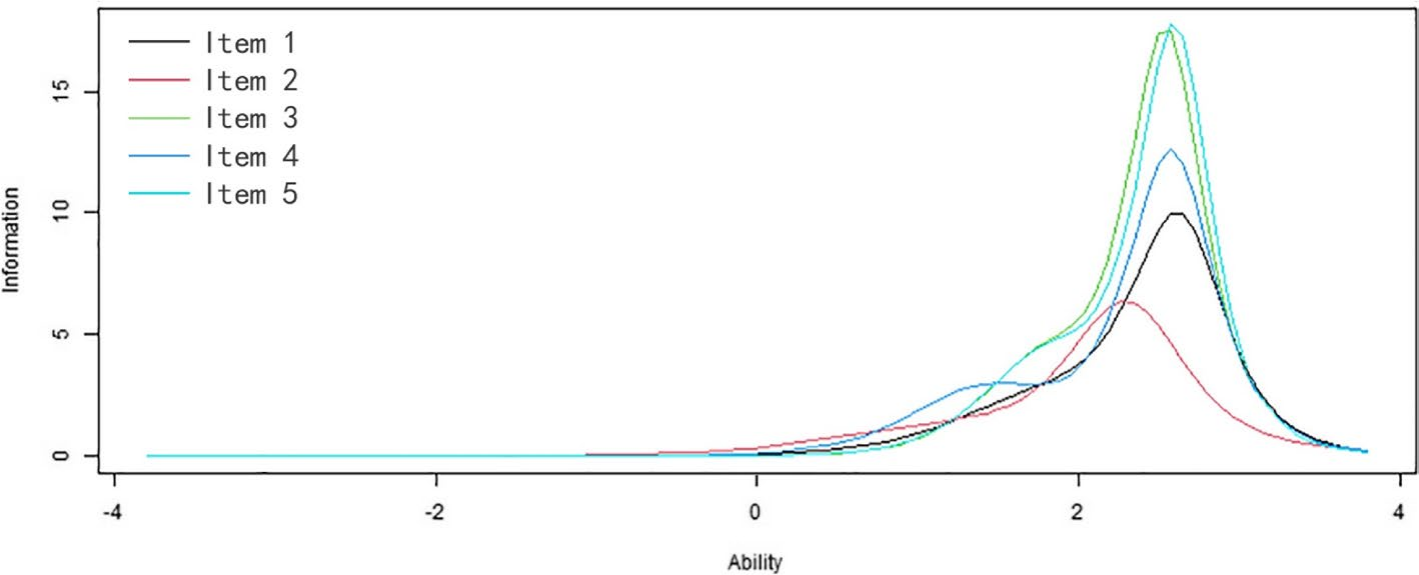
Item information curves

**Fig. 3:**
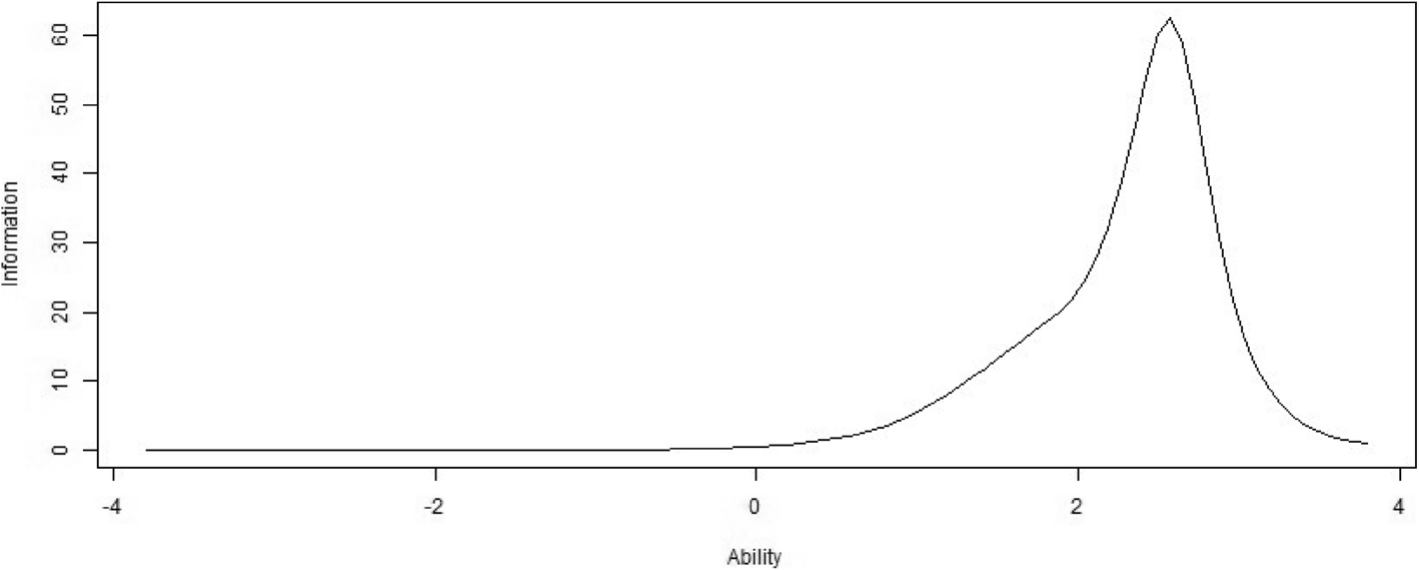
Total (scale) information curves

In the IIC, items 5, 3, 4, and 1 were the more relevant and accurate of the tool to assess the latent variable (Fig. 2). Additionally, the peak values of the maximum information skewed to the right among all of the items. The TIC of CAS had its peak value at the ability value 2.5 and displayed that the test was more precise ranging from 1 to 3.5 and most precise to the individual whose ability value was 2.5 (Fig. 3).

## Discussion

In the last two years, the CAS has been translated and validated by several researchers from different cultural backgrounds [17, 19–26]. However, there were no studies in Chinese cultural settings based on CTT. Thus, the current research aimed to present new psychometric evidence of the Chinese version according to CTT and IRT models. The test results validate that the Chinese version of the CAS has excellent psychometric properties in healthcare workers during the Omicron variants epidemic. Therefore, the CAS can be utilized for measuring the anxiety of COVID-19 in Chinese healthcare workers.

In terms of reliability, the current study also showed good results. Specifically, the split-half reliability was good. Moreover, notwithstanding the Cronbach’s α coefficient of CAS was lower than 0.900, it still indicated the CAS had excellent homogeneity considering the low number of items [55]. Compared with prior studies relating to CAS, Cronbach’s α coefficient was similar to most of them [17, 19–23, 25, 26]. The item should be removed when the Cronbach's’s α coefficient is higher than before if it is removed [56]. However, item 2 wasn’t removed, because the item-total correlation for item 2 was good, item 2 (having trouble falling or staying asleep) is the main symptom of anxiety, and Cronbach’s α coefficient marginally enhanced when item 2 was deleted. All of the item-total correlations were far more than 0.4 and good [56]. Therefore, the homogeneity of the CAS was very good. Moreover, the retest coefficient of the CAS demonstrated satisfying stability.

In the EFA model, the CAS owned only one common factor, which was not different from the original version and other versions [17, 19–23, 25, 26]. In our study, each item of the CAS owned a high load value and the communality coefficients in the single factor. Moreover, the single factor explaining variation was notably above the original version and implied satisfying construct validity [56]. Concerning the CFA, the results demonstrated convincingly that all measured values of the model fitted well without being modified, which was superior to the prior studies [24, 35]. Moreover, compared to the two dimensions, the single dimension was a better fit based on BIC [45]. The results also showed that there was strong factor loading and explanatory variance in the structural equation model, which was consistent with EFA results. As for the cross-validation, the invariance implied the CAS measuring anxiety of COVID-19 was in similar ways across ages, hospital degrees, and professional title, which was in line with prior studies [17, 24].

The results of correlation analysis implied the construct convergent validity of CAS was supported in healthcare workers. Specifically, the CAS was positively correlated with post-traumatic stress disorder, the fear of COVID, and depression, which illustrated that healthcare workers with high levels of anxiety of COVID-19 also presented high levels of post-traumatic stress disorder, the fear of COVID, and depression. These findings agreed with prior efforts and demonstrated that the construct convergent validity is excellent [17, 24, 35, 57].

As for hospital degrees, this study revealed significant differences in CAS scores. Tertiary hospitals owned elevated CAS scores compared to secondary hospitals. This illustrated the work atmosphere can play a great role in anxiety. In China, secondary hospitals mainly provide comprehensive medical services and tertiary hospitals mainly provide high-level specialized medical services [58]. In the present study, the sole tertiary hospital was the main force when the outbreak was triggered by highly contagious Omicron in the whole city. Specifically, it was responsible for the construction and management of the Fangcang shelter hospital (a temporary hospital that served to isolate patients with COVID-19). Correspondingly, a large number of healthcare workers in the tertiary hospital were drafted in to care for and treat COVID-19 patients. In terms of professional titles, intermediate grades had elevated CAS scores compared to junior and senior titles. This may be due to most of the intermediate grades, who are also parents, playing a vital role both at home and in the hospital during the pandemic. Correspondingly, their anxiety levels inevitably elevated [59].

Moreover, the CAS owns very high discrimination since all the discrimination parameters are higher than 1.70, which indicates that the CAS distinguishes the severity of anxiety of COVID-19 easily [60]. To be specific, the CAS can be easy to distinguish between the participants with high COVID-19 anxiety and those of someone with moderate or low COVID-19 anxiety. As for difficulty, the parameters demonstrated that the CAS owned acceptable difficulty. Specifically, individuals with low anxiety of COVID-19 will be inclined to select the lower response. Conversely, when an individual has a higher anxiety of COVID-19, the individual will be inclined to select a higher response [60]. As for the measurement precision, the CAS can more precisely evaluate COVID-19 anxiety in people who have moderate and high levels of the latent variable, particularly items 5, 3, 4, and 1. In the IIC, the peak value of the maximum information is skewed to the right among all of the items, which indicated that healthcare workers with high anxiety of COVID-19 have the maximum information.

### Limitations and strengths

There were several limitations in the current study. Firstly, the respondents were healthcare workers only coming from four hospitals in Anhui Province. Therefore, it is necessary to expand the sample coverage in future work. Another limitation was the unequal ratio of gender, occupation, and the number of years of employment in the sample. So, the data may apply to the others to a lesser extent. Thirdly, because of isolation and social distance during the pandemic, participants accessed the online to respond, which lead to the respondents mainly distributed among the young. It is necessary that the CAS is validated in the elder in future studies. Finally, we failed to employ a gold standard to set the optimal cutoff value, which will hinder the practical utility. Therefore, it is necessary to employ a gold standard to set the optimal cutoff value in future studies.

Notwithstanding these limitations, the current study can be considered as a groundbreaking study compared to those studies that validated CAS in China. Specifically, the current study was the first research that measured the psychometric properties of CAS employing CTT and IRT models in China. Since prior studies only applied CTT and the advantages of IRT models, this study can be considered as a valuable contribution. Moreover, some mental problems will appear following the COVID-19 pandemic, such as anxiety, and depression [35]. As noted earlier, healthcare workers owned the highest anxiety compared with other professional groups [13]. However, to date, no systematic study has tested the psychometric properties of CAS with this group in China. Thus, it is important to evaluate the anxiety of COVID-19 among healthcare workers, which is contributed to their health and the quality of health services.

In general, the results have demonstrated that the Chinese version of CAS had good content validity, construct validity, discrimination, difficulty, as well as homogeneity, and stability. Hence, the Chinese version of CAS is a suitable tool for the dysfunctional anxiety of COVID-19 for Chinese healthcare workers in the Chinese mainland.

## Conclusion

This study tested the psychometric properties of CAS in Chinese healthcare workers. The results have corroborated the Chinese version of CAS has good psychometric properties in healthcare workers during the Omicron variants epidemic, and can be used for measuring anxiety associated with COVID-19 in Chinese healthcare workers.

## Data Availability

The datasets generated and/or analyzed during the present study are not publicly available to preserve the anonymity of the participants but are available from the corresponding author at reasonable request.

## Abbreviations

COR: Conservation of Resources theory
CTT: Classical Test Theory
IRT: Item Response Theory
CAS: Coronavirus Anxiety Scale
PCL-5: Posttraumatic Stress Disorder Checklist for DSM-5
FCV-19S: The Fear of COVID-19 Scale
PHQ-2: Patient Health Questionnaire-2
CVI: Content validity index
I-CVI: Item content validity index
EFA: Exploratory factor analysis
PCA: Principal component analysis
KMO: Kaiser–Meyer–Olkin
CFA: Confirmatory factor analysis
*χ*^*2*^/*df*: Chi-square/degree of freedom
GFI: Goodness of fit index
AGFI: Adjusted goodness of fit index
RMSEA: Root-mean-square error of approximation
RMR: Root mean square residual
IFI: Incremental fit index
TLI: Tucker-Lewis index
CFI: Comparative fit index
BIC: Bayesian information criterion
MGCFA: Multiple-group confirmatory factor analysis
AIC: Akaike information criterion
GPCM: Generalized Partial Credit Model
GRM: Graduated Response Model
IIC: Item information Curves
TIC: Total (scale) information Curves
